# Insight into the Taxonomic and Functional Diversity of Bacterial Communities Inhabiting Blueberries in Portugal

**DOI:** 10.3390/microorganisms10112193

**Published:** 2022-11-04

**Authors:** Ana C. Gonçalves, Fernando Sánchez-Juanes, Sara Meirinho, Luís R. Silva, Gilberto Alves, José David Flores-Félix

**Affiliations:** 1 CICS–UBI—Health Sciences Research Centre, University of Beira Interior, 6201-506 Covilhã, Portugal; 2 CIBIT—Coimbra Institute for Biomedical Imaging and Translational Research, University of Coimbra, 3000-540 Coimbra, Portugal; 3 Instituto de Investigación Biomédica de Salamanca (IBSAL), Complejo Asistencial Universitario de Salamanca, Universidad de Salamanca, CSIC, 37007 Salamanca, Spain; 4 Departamento de Bioquímica y Biología Molecular, Universidad de Salamanca, 37007 Salamanca, Spain; 5 CPIRN-UDI/IPG—Center of Potential and Innovation of Natural Resources, Research Unit for Inland Development (UDI), Polytechnic Institute of Guarda, 6300-559 Guarda, Portugal

**Keywords:** *Vaccinium*, blueberry, bacterial diversity, plant growth-promoting bacteria, Portugal

## Abstract

*Vaccinium myrtillus* is a dwarf shrub of the *Ericaceae* family with a Palearctic distribution, associated with temperate and cold humid climates. It is widespread on the European continent; on the Iberian Peninsula it is located on Atlantic climate mountains and glacial relicts. In Portugal, we find scattered and interesting populations; however, the majority of them are threatened by climate change and wildfires. Given that, the objective of this study is to determine the rhizospheric and root bacterial communities of this plant in the southernmost regions, and, consequently, its potential range and ability to be used as a biofertilizer. In this work, metabarcoding of 16S rRNA gene showed that the endophytic bacterial diversity is dependent on the plant and selected by it according to the observed alpha and beta diversity. Moreover, a culturomic approach allowed 142 different strains to be isolated, some of them being putative new species. Additionally, some strains belonging to the genera *Bacillus, Paenibacillus, Pseudomonas, Paraburkholderia*, and *Caballeronia* showed significant potential to be applied as multifunctional biofertilizers since they present good plant growth-promoting (PGP) mechanisms, high colonization capacities, and an increase in vegetative parameters in blueberry and tomato plants.

## 1. Introduction

The *Vaccinium* genus includes more than 450 different species of shrubs, ranging from just a few centimeters to several meters in height. They are mainly reproduced by underground stolons, and less by seeds [1]. Although they have a circumboreal distribution, most of the species are restricted to temperate and humid environments, usually cold, and to soils with a high water availability [1,2,3]. Additionally, this genus prefers substrates with pH values between 4.5 and 5.5 [2]. Among the species of this genus, those that are more commonly cultivated are *V. myrtillus*, *V. corymbosus*, *V. macrocarpum*, and *V. vitis-idaea*. Their fruits exhibit a notable antioxidant capacity, mainly due to the high concentration of phenolic compounds [4]. 

In the Iberian Peninsula, the occurrence of this genus is determined by the species *V. myrtillus*, *V. uliginosum*, and *V. vitis-idaea.* The development of this genus is associated with regions dominated by an Atlantic climate and high rainfall, mild temperatures, humid summers, and relatively abundant rainfall distributed throughout the year [5]. *V. myrtillus* is the most widespread species found in the understory of oak and coniferous forests, having outstanding ecological importance here since its fruits serve as food for numerous species, some of which are threatened with extinction, such as the Cantabrian capercaillie (*Tetrao urogallus* subsp. *cantabricus*) [6,7]. As glacial relicts, isolated populations of this species are associated with the mountain systems in the center and south of the Iberian Peninsula [8]. Focusing on the mainland of Portugal, we find populations of *V. uliginosum* only in the highest areas of Serra da Estrela, with fragmented populations and doubtful survival [9]. However, *V. myrtillus* is more abundant in the mountains of northern Portugal, such as Serra da Freita and Serra do Marão; even so, they also have fragmented populations in Serra da Estrela.

However, they are highly endangered due to climate change, which predicts a reduction in the average rainfall in this region, as well as anthropogenic threats from land use, also owing to the large number of fires that lead to habitat fragmentation and destruction of the soil on which this plant spreads [5,9]. In turn, the disappearance of native blueberry populations in Portugal has coincided with a boom in the cultivation of blueberries for agriculture, mainly *V. corymbosum*, but also some varieties of *V. myrtillus* [8]. For this reason, the development of bacterial biofertilizers is a tool to be considered. These microorganisms are commonly selected from rhizospheric or endophytic populations [10]. Plants are capable of enriching and selecting rhizospheric populations of bacteria by producing root exudates [11]. Thus, only some taxa are capable of colonizing the interior of the plant, called endophytes [12]. The selection of usable microorganisms goes through isolation and selection based on their plant growth-promoting (PGP) mechanisms. These mechanisms can be direct, such as nitrogen fixation, phosphate solubilization, siderophore production, or phytohormone synthesis; or indirect, such as the formation of biofilms or the production of lytic enzymes, among others [13]. For this purpose, the cultivation of microorganisms is essential, with bacteria being a wise choice due to the large number of PGP mechanisms they can produce. Most studies have focused on ericoid mycorrhizal populations [14,15], and bacterial populations have only been studied in regions of northern Europe where this plant is widespread and a part of forest ecosystems [16]. However, these populations in the Iberian Peninsula have not been studied so far. Moreover, knowledge about them may help us to design strategies for the conservation, propagation, and development of biofertilizers for blueberry cultivation in the west of the Iberian Peninsula, where it is a booming crop. Therefore, the objective of this work is to analyze, for the first time, the bacterial populations associated with *V. myrtillus* in Portugal using metabarcoding and culturomic techniques, and to determine their potential for the development of effective biofertilizers for this plant genus.

## 2. Materials and Methods

### 2.1. Metabarcoding of V. myrtillus Populations in Portugal

Samples of *V. myrtillus* plants were collected from the main representative locations in Portugal: Serra do Marão (41°15′55.6′′ N 7°51′20.4′′ W), Serra da Freita (40°51′53.1′′ N 8°16′47.1′′ W), and Serra da Estrela (40°21′50.9′′ N 7°38′13.0′′ W). The rhizospheric material was separated from the root. We collected three samples of rhizospheric soil and three samples of root for each location. Afterwards, samples (roots and rhizospheric soil) were transported at 4 °C to the laboratory facilities, and then immediately frozen in liquid nitrogen and stored at –80 °C until processing. Total root DNA extraction to analyze the endophytic bacterial communities was carried out using the NZY Plant/Fungi gDNA Isolation Kit (NZY Tech, Lisbon, Portugal) according to the manufacturer’s instructions. To that end, the roots were firstly sonicated three times for 30 s and washed 10 times with distilled water to remove the populations that adhered to the surface. Subsequently, the DNA extraction from rhizospheric bacterial populations was performed using the NZY Plant/Fungi gDNA Isolation Kit according to the manufacturer’s instructions. The V3–V4 region was selected to characterize the bacterial population associated with *V. myrtillus*. For library preparation, a fragment of the bacterial 16S rRNA region of approximately 420 bp was amplified with the primers Bakt 341F (5′ CCT ACG GGN GGC WGC AG 3′) and Bakt 805R (5′ GAC TAC HVG GGT ATC TAA TCC 3′) [17]. Illumina sequencing primers were appended to these primers at their 5′ ends.

PCRs were performed in a final volume of 25 µL, containing 2.5 µL of template DNA, 0.5 µM of the primers, 6.5 µL of Supreme NZYTaq 2x Green Master Mix (NZYTech), and ultrapure water to make up the final volume. The reaction mixture was incubated as follows: an initial denaturation at 95 °C for 5 min, followed by 25 cycles of 95 °C for 30 s, 50 °C for 45 s, 72 °C for 45 s, and a final extension step at 72 °C for 7 min. The oligonucleotide indices that are required for multiplexing different libraries in the same sequencing pool were annealed in a second round of PCR with identical conditions, but only involving five cycles and using 60 °C as the annealing temperature [18].

A negative control with no DNA (BPCR) was included in each PCR round to check for contamination during library preparation. Libraries were purified using the Mag-Bind RXNPure Plus magnetic beads (Omega Bio-tek, Norcross, GA, USA), according to the manufacturer’s instructions. Then, they were pooled in equimolar amounts according to the quantification data of the Qubit dsDNA HS assay (Thermo Fisher Scientific, Waltham, MS, USA). The pool was sequenced in a fraction of a MiSeq PE300 run (Illumina, San Diego, CA, USA). The data obtained were deposited in the GenBank under the Bioproject PRJNA786857, SRA numbers SRX13339950 to SRX13339967.

The quality of the FASTQ files was checked using FastQC software and summarized using MultiQC [19]. The obtained 16S amplicon reads were processed using QIIME2 (version 2020.2) [20]. DADA2 [21] implemented in QIIME2 was used to remove the PCR primers, filter the reads according to their quality, denoise, merge the reads, remove the chimeric reads, and cluster the resulting sequences into amplicon sequence variants (ASVs). The EzBioCloud pipeline was used to estimate alpha and beta diversity using PKSSU4.0 and open reference UCLUST_MC2 for OTUs picking at a 97% cutoff [22]. Variance of detected taxa was performed with BioVinci (BioTuring, San Diego, CA, EEUU), using hierarchical clustering by Euclidean linkage as a parameter to compare populations between samples. Significant differences between taxonomic distribution at the genus level of bacterial communities retrieved from each sample was evaluated by means of the software Past 4 (https://past.en.lo4d.com/windows, accesed on 16 June 2022), and the Anova test, Mann–Whitney pairwise comparison; Bonferroni-corrected (*p* < 0.05). Beta diversity was analyzed using the *PERMANOVA* test implemented on MTP tool of EzBioCloud [22]

### 2.2. Isolation of Bacteria from V. myrtillus

The isolation of bacteria present in the rhizospheric and endophytic populations of *V. myrtillus* in Portugal was performed by isolating them in TSA [23], YMA [24], MRS [25], and a nitrogen-free medium [26] from the same sites for which the characterization was performed. Two different strategies were followed depending on whether they corresponded to the rhizosphere or the root material. For the isolation of bacteria from rhizosphere material, 1 g of soil was taken in triplicate and dissolved in 99 mL of sterile deionized water, shaken for 1 h at 120 rpm and 28 °C. Subsequently, serial dilutions were made and seeded in the indicated media. For isolation of endophytic bacteria from root samples, 1-cm-long pieces of blueberry root were collected from each site, washed three times with sterile deionized water, and sterilized by immersion in ethanol (70%, *v*/*v*) for 30 s and sodium hypochlorite (2%, *v*/*v*) for 3 min, and then washed five times with sterile deionized water. A sample of the last wash water was taken and cultured in TSA medium as a negative sterilization control. Roots were crushed in PBS (pH 7.4), and serial dilutions were prepared in sterile deionized water for subsequent seeding in the indicated media. Petri dished were incubated at 28 °C for 7 days. Both procedures were performed in triplicate for each site. Isolates were grown until pure cultures were obtained.

### 2.3. Identification and PGP Characterization of Isolated Bacterial Strains

#### 2.3.1. MALDI-TOF

The sample preparation and MALDI-TOF MS analysis were performed as previously described [27], using a saturated matrix solution of α-HCCA (Bruker Daltonics, Bremen, Germany), dissolved in 50% acetonitrile and 2.5% trifluoroacetic acid. Amounts of biomass between 5 and 100 mg were used to obtain the spectra indicated by the manufacturer. The calibration mass was conducted based on the Bruker Bacterial Test Standards (BTS), using masses as averages: RL36, 4365.3 Da; RS22, 5096.8 Da; RL34, 5381.4 Da; RL33meth, 6255.4 Da; RL29, 7274.5 Da; RS19, 10,300.1 Da; Rnase A, 13,683.2 Da; and myoglobin, 16,952.3 Da. 

The score values proposed by the manufacturer are as follows: a score value between 2.3 and 3.00 indicates highly probable species identification, a score value between 2.0 and 2.299 indicates secure genus identification and probable species identification, a score value between 1.7 and 1.999 indicates probable genus identification, and a score value <1.7 indicates no reliable identification. 

Cluster analysis was performed based on a comparison of strain-specific main spectra, created as described above. The dendrogram was constructed by the statistical toolbox of Matlab 7.1 (MathWorks, Inc., Natick, MS, USA) integrated with MALDI Biotyper 3.0 software. The parameter settings were: “Distance Measure = Correlation average” and “Linkage = Complete”. The linkage function was normalized according to the distance between 0 (perfect match) and 1000 (no match).

#### 2.3.2. Identification Based on Sequencing of 16S rRNA 

The amplification and sequencing of the *16S rRNA* gene were carried out as indicated previously [28] using the primers *27F* (5′-AGAGTTTGATCCTGGCTCAG-3′) and *1522R* (5′ AAGGAGGTGATCCANCCRCA 3′). The sequences obtained were compared with those from GenBank using the BLASTN program, selecting “type strains” as phylum to perform the search [29]. Obtained sequences were deposited in GenBank. Accession numbers are included in Table 1.

#### 2.3.3. Analysis of the Plant Growth-Promoting (PGP) Potential of Isolated Strains

The solubilization of insoluble phosphate was analyzed on Pikovskaya plates containing 2% CaHPO_4_, Ca_3_(HPO_4_)_2_ and hydroxyapatite, and incubated for 15 days at 28 °C. Standard determination was performed using a phosphate solubilization ratio that was calculated as the ratio between the halos around the colony with respect to colony size [13]. Siderophore production was evaluated in M9-CAS-AGAR [13] modified with the addition of a cationic solvent, HDTMA, which stabilizes the Fe–CAS complex and allows for the detection of siderophore production by the appearance of an orange halo around colonies. Indole acetic acid (IAA) production was evaluated in a JMM medium [23] supplemented with 107 mg/L of tryptophan. After seven days of incubation, the medium supernatants were recovered by centrifugation at 5000× *g* and filtered using 0.22-µm Millipore filters (Millipore Co., Burlington, MA, USA). Then, 1 mL of Salkowski reagent was added to 2 mL of supernatant, and the red color formed was measured by spectrophotometry at 550 nm using an ATI Unicam 8625 spectrometer (Mattson, Madison, WI, USA). The production of IAA for the selected isolates was quantified by an HPLC-DAD method. The analysis was performed using an LC-2010A HT Liquid Chromatography system coupled with an SPD-M20A diode-array detector, automatically controlled by LabSolutions 5.52 data acquisition software (Shimadzu, Kyoto, Japan). Chromatographic separation was performed at 30 °C on a reversed-phase column (C18, 3 µm particle size, 55 mm× 4 mm) protected by a reversed-phase precolumn (C18, 5 µm particle size, 4 mm × 4 mm), both of LiChroCART^®^ Purospher^®^ STAR models purchased from Merck (Darmstadt, Germany). Isocratic elution was achieved at a 1 mL/min flow rate using a mobile phase composed of 50% acetonitrile and 50% water with 0.02% acetic acid. 

### 2.4. In-Plant Evaluation of PGP Ability 

For plant colonization assays, 30 seeds of each plant were surface sterilized. *V. myrtillus* seeds were surface sterilized by immersion in 70% ethanol for 30 s, followed by soaking in an aqueous 5% sodium hypochlorite solution for 2 min. *Lycopersum sculentum var. Roma* seeds were surface sterilized by immersion in ethanol (70%, *v*/*v*) for 30 s, followed by soaking in an aqueous sodium hypochlorite solution (5%, *v*/*v*) for 5 min. Seeds were then washed six times with sterile water and germinated in water–agar plates overlaid with Whatman number 1 sterile paper wetted with sterile water. The plates were placed in darkness in a growth chamber at 24 °C until the seedling roots emerged. Next, seeds were transferred to Rigaud & Puppo Agar plates overlaid with Whatman number 1 sterile paper. Each seed was inoculated with 300 µL of a suspension of selected strains with a concentration of 10^7^ UFC/mL. Data were collected 7 and 14 days before inoculation. Number of secondary roots and shoots and root lengths results were subjected to ANOVA analyses followed by post hoc Tukey’s t-tests to identify inoculation treatments with means significantly higher than those of the control. All statistical analyses were performed using the statistical package GraphPad Prism 8 (GraphPad Software, San Diego, CA, USA)

### 2.5. Colonization Ability of Isolated Bacteria

Two different protocols were used to detect bacteria attached to blueberry roots. The pHC60 plasmid [30], harboring a gene expressing a green fluorescent protein, was introduced into *Pseudomonas, Caballeronia*, and *Paraburkholderia* strains by transconjugation. These strains were grown for 48 h at 28 °C on TSA plates supplemented with tetracycline at 10 µg/mL, and then washed twice and resuspended in sterile water to a final concentration of 10^8^ CFU/mL. The second protocol was applied to *Bacillus* and *Paenibacillus* strains. To that, an inmunolocation protocol was applied following the whole-plant preparation. Roots were removed from the plant and fixed overnight in formaldehyde (4%, *v*/*v*). Then, they were blocked with a solution of powdered milk (3%, *w*/*v*) and Triton-X (0.03%, *v*/*v*) dissolved in PBS (pH 7.4) for 1 h at room temperature. Roots were then washed three times with PBS and incubated with a primary antibody (Gram Positive Marker Antibody, Santa Cruz Biotechnology, Dallas, TX, USA) overnight at 4 °C. Next, roots were washed three times with PBS and mixed with a secondary antibody (m-IgGkappa BP-CFL 488, Santa Cruz Biotechnology). They were then washed with PBS and stored submerged at 4 °C and protected from light until use.

For plant colonization studies, 30 seeds of *V. myrtillus* were surface sterilized by immersion in ethanol (70%, *v*/*v*) for 30 s, followed by immersion in an aqueous sodium hypochlorite solution (5%, *v*/*v*) for 3 min. Seeds were then washed six times with sterile water, and germinated in water–agar plates lined with sterile paper Whatman number 1 moistened with sterile water. The plates were kept in the dark in a growth chamber at 24 °C until the roots of the seedlings were 1–2 cm long. Then, blueberry seedlings were inoculated with 1 mL of this suspension. The seedlings were kept in the dark and the roots were observed under a light microscope 7 and 14 days after inoculation. To remove unattached bacteria, the roots were carefully washed three times with sterile distilled water before microscopic observation. Uninoculated blueberry roots were included in the experiment as negative controls. Fluorescence microscopy was performed with a Zeiss Axio Imager A1 (Zeiss, Jena, Germany), and excitation of the green fluorescent proteins and Alexa-488 fluorophore bound to the secondary antibody was performed with a mercury lamp. Root cells were stained with 10 µM of calcofluor white (Sigma-Aldrich, San Luis, MI, USA). 

## 3. Results

### 3.1. The Composition of Bacterial Communities Associated with V. myrtillus in Portugal

**Figure 1:**
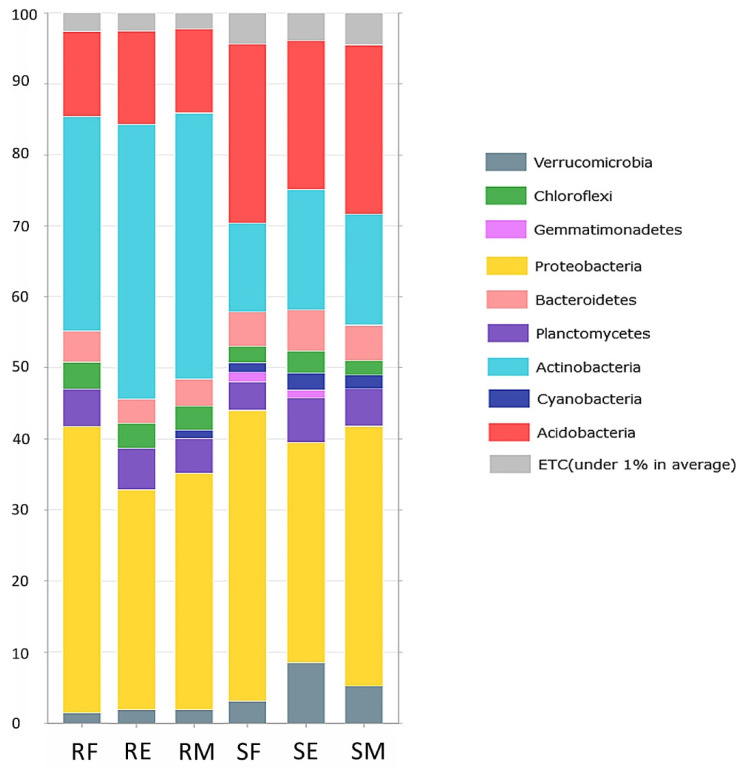
Average of phylum composition of obtained sequence on each sampling location. Abbreviations: RF—root endophytes Serra da Freita, RE—root endophytes Serra da Estrela, RM—root endophytes Serra do Marão, SF—soil Serra da Freita, SE—soil Serra da Estrela, SM—soil Serra do Marão. ETC: et cetera, representing phyla whose average percentage was under 1%.

The dataset contained a total of 12,916 features encompassed by 1,489,679 filtered reads (1,814,440 before filtered) spread across 18 samples, with a median frequency of 82,759 reads/sample. Complete sequencing statistics are included in Table S1. Metagenomic analysis of Portuguese *V. myrtillus* populations showed that the rhizospheric communities were dominated by the phylum Proteobacteria and Acidobacteria in similar percentages, alternatively at each of the sampled sites, followed by Actinobacteria. While the percentage of readings attributable to Actinobacteria increased, a significant decrease in the phylum Acidobacteria was observed. The phylum Cyanobacteria was present in all rhizospheric communities but appeared only in the endophytic communities of Serra do Marão. The phylum Gemmatimonadetes was found only in the rhizospheric communities of Serra da Freita and Serra da Estrela, and the phylum Verrucomicrobia was particularly abundant (7%) compared to the rest of the analyzed communities. Therefore, it is worth noting that the percentage of phyla that did not reach 1% abundance was twice as high in the rhizospheric communities as in the endophytic communities, ranging from 2.4% to 5% (Figure 1).

**Figure 2:**
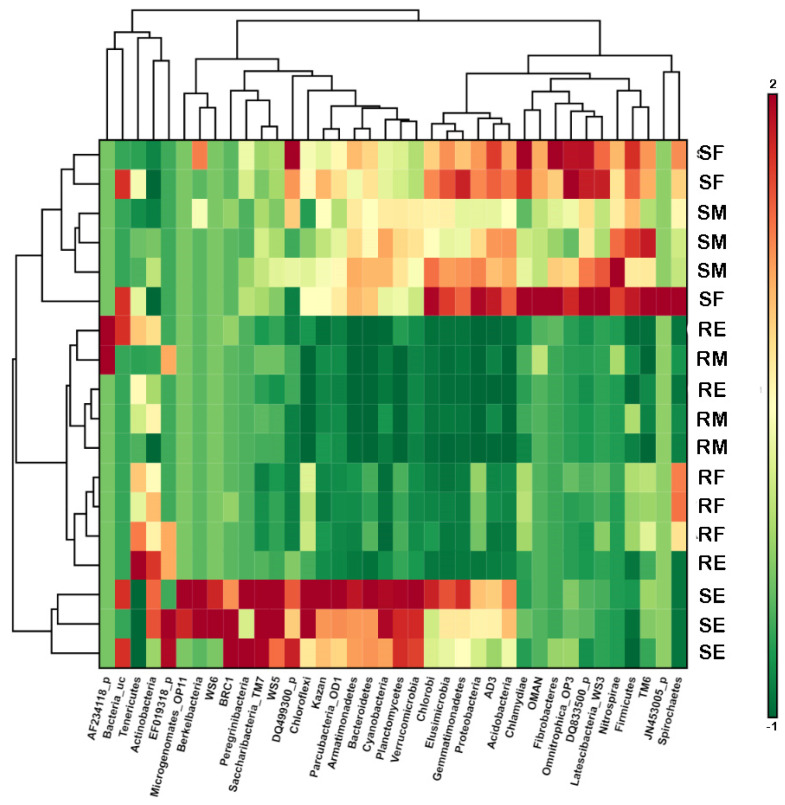
Standardized abundance of different phyla in each sample using hierarchical clustering by Euclidean linkage as parameters. Green colors indicate reduction in abundance and red colors indicate increase in abundance with respect to normalized abundance of each phylum. Abbreviations: RF—root endophytes Serra da Freita, RE—root endophytes Serra da Estrela, RM—root endophytes Serra do Marão, SF—soil Serra da Freita, SE—soil Serra da Estrela, SM—soil Serra do Marão.

Observing the heatmap (Figure 2), it is possible to see the influence of a more Atlantic climate on bacterial communities present in the rhizospheres of *V. myrtillus.* A similar proportion and distribution of the obtained sequence at phyla level, and a reduced abundance of Actinobacteria, Tenericutes, and Berkelbacteria, were found. The *V. myrtillus* communities of Serra da Freita showed an increase in the abundance of Chlamydiae, Acidobacteria, Fibracteres, Omnitrophica_OP3, TM6, Nitrospirae, and Firmicutes with respect to the rest of the communities analyzed. On the other hand, the rhizospheric communities of *V. myrtillus* from Serra da Estrela exhibited a discordant community distribution compared with the previous ones, with enrichment in Actinobacteria, Berkelbacteria, Peregrinibacteria, Bacteroidetes, and Cyanobacteria. This could be due to the peat bog environments. Regarding the endophytic communities of *V. myrtillus*, similar behavior was observed in all the communities, showing a clear example of modulation and selection of the endophytic communities by the plant. In this case, the enrichment in the populations of Actinobacteria and Tenericutes, and of Spirochaetes, was observed only in the endophytic populations of Serra da Freita.

**Figure 3:**
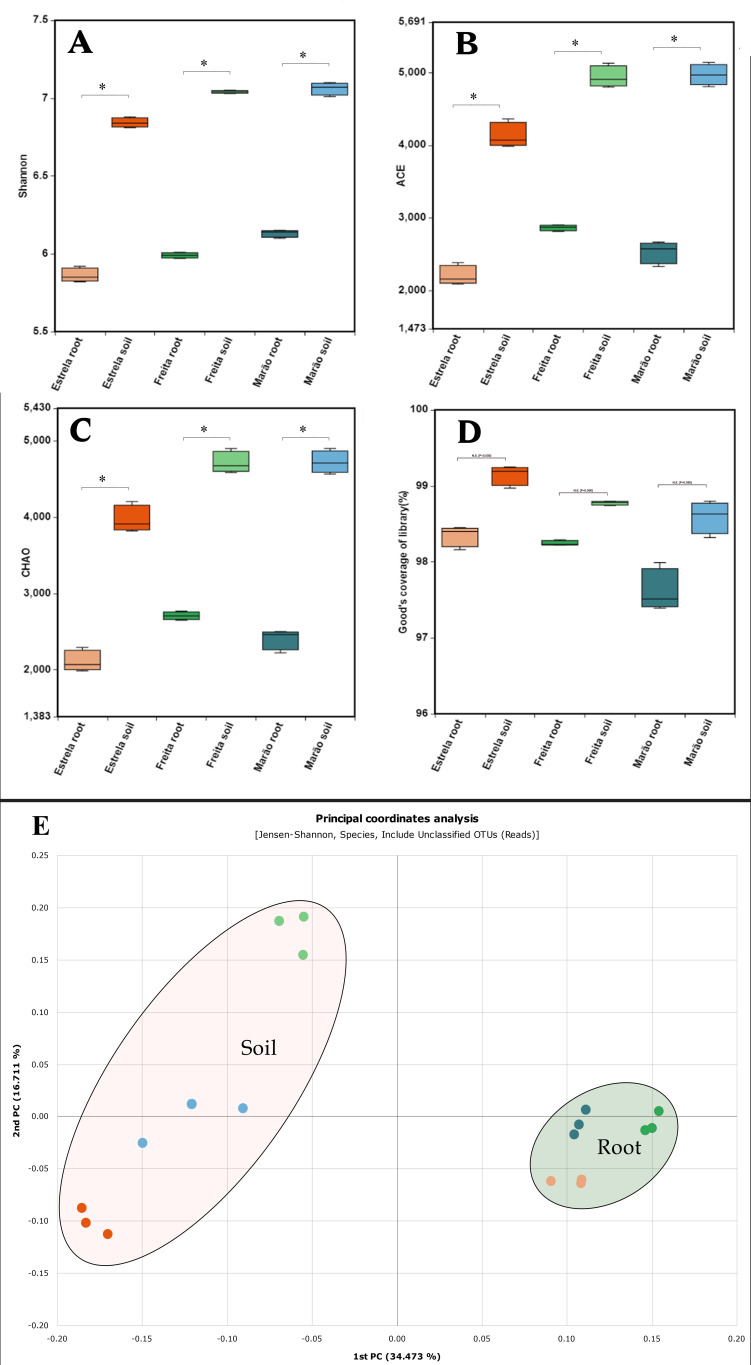
Analysis of the α- and β-diversity of bacterial communities. (**A**) Shannon–Wiener index, (**B**) ACE index, (**C**) Chao index, (**D**) Good’s coverage index, and (**E**) PCoA showing the distribution of bacterial communities based on their species composition. *: indicates significant differences (Anova; Mann–Whitney pairwise comparison; Bonferroni-corrected *p* values; *p* < 0.05).

We analyzed the α-diversity according to the Shannon–Wiener index, which showed that the rhizospheric communities present much higher diversity than those detected in the endophytic ones. The rhizospheric communities of Serra da Freita and Serra do Marão displayed values of 7 and 7.2, respectively (Figure 3a), while the rhizospheric communities of Serra da Estrela had a value of 6.7. In this regard, the endophytic communities of *V. myrtillus* from Serra do Marão presented the most diverse values (6.2), followed by Serra da Freita (6.1) and Serra da Estrela (5.9). On the other hand, the β-diversity analysis showed a scattering of rhizospheric communities among the sites, while the endophytic communities presented a similar trend in the three communities analyzed (Figure 3a). These values revealed the selective effect of the root on the rhizospheric communities in terms of both quantity and diversity, being in accordance with a Good’s coverage index greater than 98% in all cases, except the samples from the root and Serra do Marão (Figure 3d). To illustrate the influence of the plant on the microbiomes, a PCoA analysis was performed, followed by the calculation of the beta diversity of blueberry populations from Portugal, according to the Janson–Shannon index. Although they presented higher dispersion, it was observed that the rhizospheric communities created a clearly separated cluster from the rhizospheric communities, grouped in a different and compact group, showing that the endophytic communities are influenced and selected by the plant supported by a PERMANOVA pairwise test (PERMANOVA, pseudo-F  =  7.894; *p*  =  0.001; *q =* 0.001) (Figure 3e).

### 3.2. Culturomic Analysis of the Bacterial Communities of Portuguese Blueberries

For subsequent use, the design of biofertilizers requires the isolation of microorganisms. The isolation of bacteria was carried out using different TSA, YMA, MRS, and Free-N mediums. A total of 142 isolates were obtained from the three locations studied from rhizosphere and root samples (Table S2). An identification using MALDI-TOF allowed the identification of 35.7% of the isolates with a score greater than 2000, being in agreement with a secure identification at the genus level. Furthermore, 20.8% of the isolates were identified at probable genus level (scores between 1.700 and 2.000), whereas 43.5% could not be assigned to any genus (values lower than 1.700). It was observed that many pathogens, such as *Staphylococcus capitis* and *Serratia liquefaciens*, were identified with scores higher than 2.3, as well as some strains belonging to the genera *Bacillus* and *Pseudomonas*.

**Figure 4:**
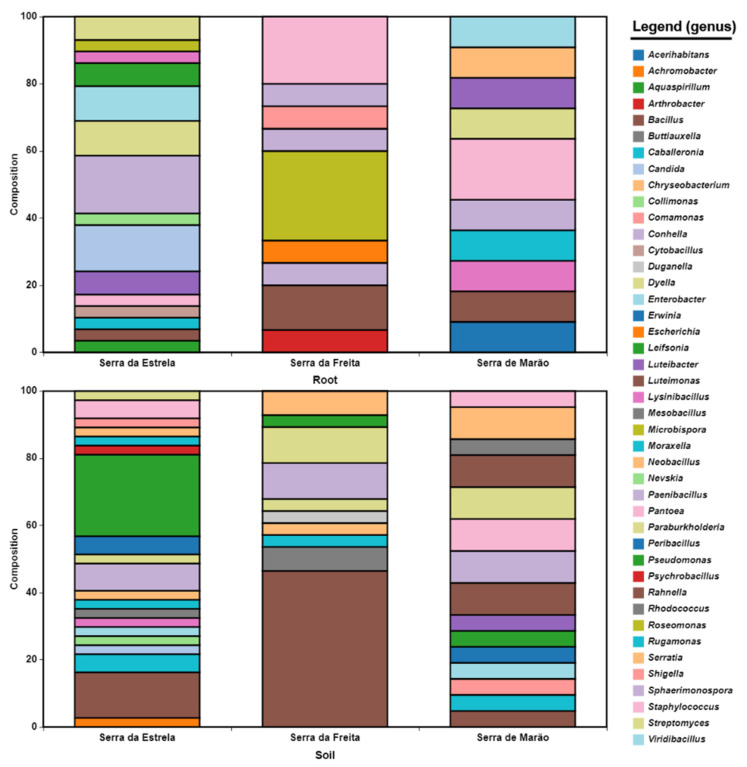
The composition of the bacterial culturome associated with Portuguese blueberries at the genus level.

To complete the identification of the isolated and cultured strains, the 16S ribosomal gene was sequenced in all isolates whose identification at the species level was doubtful (score: 2.0–2.3) or that had a lower score (pathogenic genera were excluded from the study). The results of the identification are shown in Table S2, which also includes the accession number of the sequences obtained and already deposited in the NCBI database. In this way, most isolates had an identification percentages above 99%. Even so, some strains had the potential to be new species, such as VMSES03, VMSES38, VMSES63, VMFR46, or VMMAR13, since they exhibited percentages below 99%. Thus, the number of genera detected in the rhizospheres of the three sites was higher than that detected in the endosphere of the roots, with the genus *Bacillus* and other related genera, such as *Mesobacillus, Neobacillus*, or *Psychrobacillus,* dominating in Serra da Estrela and Serra da Freita (Figure 4). In agreement with the metagenomic analyses, endophytic *Actinobacteria* were represented by the genera *Streptomyces*, *Microbispora*, and *Arthrobacter*. Another genus that showed different behavior was the genus *Pseudomonas*, which occurred in the rhizospheres of the three sites but was isolated only in the endosphere of the Serra da Estrela site. Thus, other genera such as *Caballeronia* and *Paraburkholderia,* although they were also frequently isolated from the rhizosphere, appeared to be more abundant in the endosphere. It also appeared that the Serra da Estrela endosphere had a more diverse culturome; even so, many of the genera were represented by only a single strain (Figure 4).

### 3.3. Evaluation of the PGP Potential of the Isolated Strains

**Figure 5:**
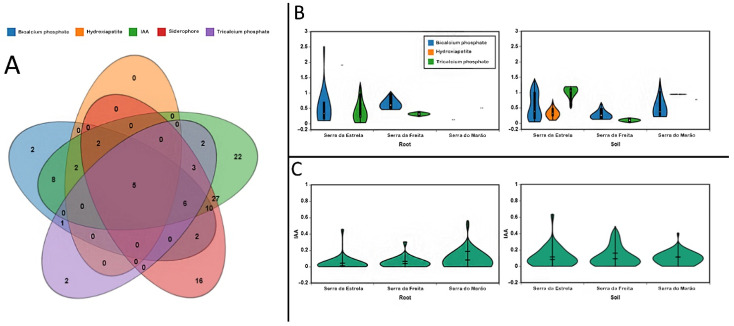
PGP mechanisms of the isolated and nonpathogenic strains. (**A**) Venn diagram of the PGP mechanisms of studied strains, (**B**) phosphate solubilization, and (**C**) IAA production.

Subsequently, screening was performed to determine the PGP potential of the isolated bacteria that had not presented pathogenic potential in the complementary identification by means of MALDI-TOF and sequencing of the 16S ribosomal gene. Most of the isolates demonstrated some PGP mechanism (Table S2). The most abundant PGP mechanism was the production of IAA, present in 60% of the isolates, followed by the production of siderophores, and by the solubilization of bicalcium phosphate, tricalcium, and hydroxyapatite (Figure 5). Only five of the isolates exhibited the production of the five PGP analyzed mechanisms, and the most common combination of mechanisms was the production of siderophores and the synthesis of IAA. Phosphate solubilization was more common among the isolates from the Serra da Estrela location, with strains such as VMSES31 showing an outstanding capacity for it. In addition, phosphate solubilization was more common among rhizosphere isolates than endophytic isolates. It is also noteworthy that the isolates from Serra do Marão presented the most discrete values of solubilization of the three analyzed phosphate sources (Figure 5b). In turn, the production of IAA, detected in 84 of the isolates, seems to follow a similar behavior, i.e., a higher production capacity of the rhizosphere isolates than the endophytic isolates, in the three studied locations (Figure 5c).

In this way, selected strains presented the strongest, and the most balanced and optimized combination of PGP mechanisms: *Psychrobacillus glaciei* VMSES02, *Pseudomonas frederiksbergensis* VMSES14, *Paraburkholderia domus* VMSES31, *Pseudomonas koreensis* VMSES32, *Paenibacillus* sp. VMSES63, *Paenibacillus amylolyticus* VMSES74, *Caballeronia sordidicola* VMMAR66, *Bacillus aerius* VMFR04B, *Bacillus altitudeinis* VMFR45, *Paenibacillus* sp. VMFR46, *Caballeronia jiansuensis* VMFR53, *Pseudomonas salomonii* MIRT15, *Paenibacillus pini* MIRT26, and *Pseudomonas allii* MIRT39A. The ability of the strains to produce IAA was quantitatively evaluated by HPLC (Table 1). First, the values obtained differed from those obtained by the semiquantitative technique, despite showing a similar trend. Thus, the strains *Pseudomonas* MIRT15 and MIRT39A produced the highest concentration (38.37 and 26.57 µg/mL, respectively). Most of the other selected strains produced rather discrete concentrations, between 4 and 0.5 µg/mL.

### 3.4. Analysis of PGP on Plant Assays

The ability of the selected PGP strains to promote plant growth was evaluated in blueberry and tomato seedlings. The first one was used as the plant under study, and the second one as a model plant to study the capacity to promote plant growth. The PGP capacity in the plant was evaluated on days 7 and 14 by measuring the size of the aerial part, the root length, and the number of secondary roots. The obtained results are shown in Table 2. Tomato inoculation showed that, after one week of inoculation, treatments with VMFR45, VMFR46, and VMSES31 strains resulted in an increase in root length and in the number of secondary roots. It is worth highlighting that the treatment with the VMSES02 strain produced an increase in the number of secondary roots of 280%. The treatments with VMFR45 and VMFR46 strains, followed by VMSES14 strain, generated the greatest increase in the length of aerial parts. A remarkable fact is that the MIRT15, MIRT26, and MIRT39A strains, despite presenting noteworthy in vitro PGP mechanisms (Table 1), did not generate a positive response in the inoculated plants, even reducing the number of secondary roots, as in the case of the MIRT15 strain. The data obtained on day 14 after inoculation showed a similar trend, where the treatments with the VMFR45 and VMFR46 strains presented the best values in promoting aerial and root length. The treatment with VMSES31, VMSES32, and VMSES74 strains increased the root size. In the case of the number of secondary roots, those treatments multiplied by 3- and 4-fold the number of secondary roots with respect to the negative control without inoculation.

Secondly, the ability of the strains to promote the development of blueberry seedlings was studied. It was observed that, after seven days, no statistically significant differences in the aerial and root lengths were found (Table 2). Only the treatments with VMFR53, VMSES14, and VMSES74 strains produced statistically significant increases in the number of secondary roots with respect to the negative control after seven days of inoculation. The results obtained 14 days after inoculation of blueberry seedlings showed outstanding results in terms of improving the root and aerial lengths compared with the negative control (Table 2). In fact, the treatment with 12 different strains produced increases in root length, while 11 strains showed increases in aerial length. Moreover, treatments with the strains VMFR46, VMFR53, VMSES14, VMFR45, VMSES74, and VMSES31 produced the most notable increments in aerial length, with statistically significant increases of 129%, 114%, 114%, 108%, 106%, and 104% respectively. Regarding the treatments with VMFR46 and VMFR04B strains, they promoted increases of 63% and 40%, respectively; however, no statistically significant differences were observed in the number of secondary roots in the inoculated seedlings with respect to the negative control (Table 2).

### 3.5. Analysis of Root Colonization in Blueberry Seedlings

**Figure 6:**
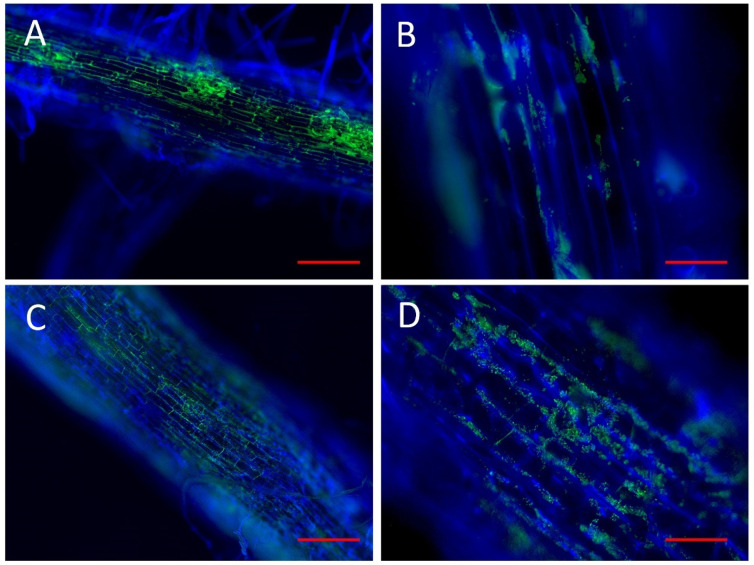
Fluorescence optical micrographs of roots of the blueberry seedlings colonized by *Paraburkholderia domus* VMSES31 ((**A**)-bar = 500 µm, (**B**)-bar = 75 µm), *Caballeronia jiansuensis* VMFR53 ((**C**)-bar = 500 µm, (**D**)-bar = 75 µm). In green are the bacterial cells of the studied strains and in blue the root cells stained with calcofluor white.

The strains that provided the best response in tomatoes and blueberries were selected to analyze their ability to colonize the blueberry rhizosphere. The selected strains were VMSES31, VMSES74, VMFR45, VMFR46, and VMFR53. Two different techniques were used to study their colonization capacity, depending on their phylogenetic affiliation. Strains VMSES31 and VMFR53, identified in the *Paraburkholderia* and *Caballeronia* genera, were located by inserting the plasmid phC60 encoding a GFP. The VMFR45, VMFR46, and VMSES74 strains, respectively, identified in the *Bacillus*, *Paenibacillus*, and *Paenibacillus* genera, were located by immunohistochemistry using the Gram-positive marker antibody (Santa Cruz Biotechnology). Strains VMSES31 and VMFR53 showed similar colonization patterns, covering the root surface (Figure 6a,c). Comparing them, VMSES31 showed more surface colonization (Figure 6a), but a similar pattern was observed following root epithelial cell unions (Figure 6b,d). 

**Figure 7:**
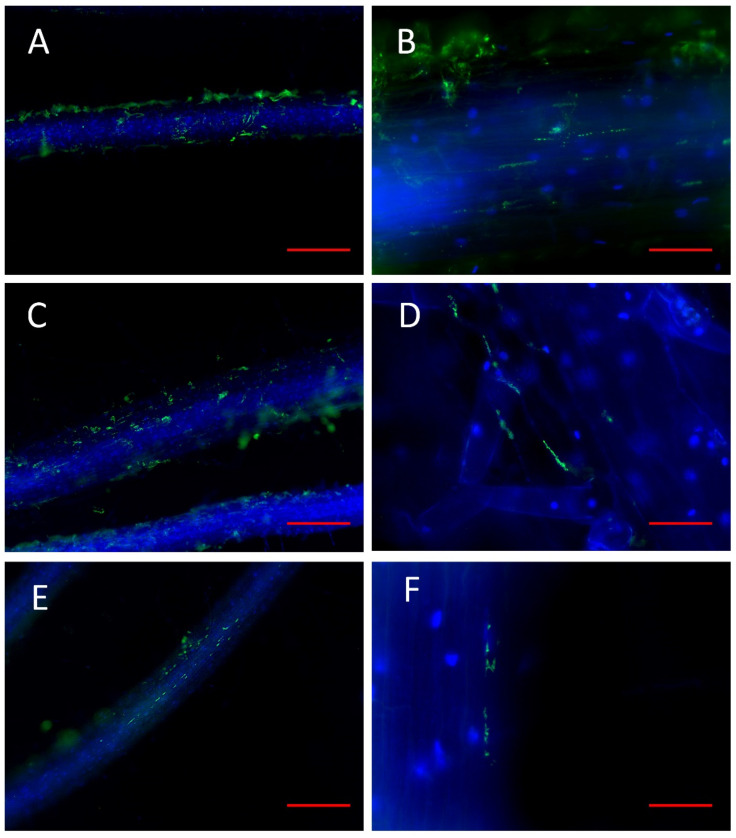
Fluorescence optical micrographs of roots of the blueberry seedlings colonized by *Bacillus altitudeinis* VMFR45 ((**A**)-bar = 500 µm, (**B**)-bar = 75 µm), *Paenibacillus* sp. VMFR46 ((**C**)-bar = 500 µm, (**D**)-bar = 75 µm) and *Paenibacillus amylolyticus* VMSES74 ((**E**)-bar = 500 µm, (**F**)-bar = 75 µm). In green are the immunolocated cells of the studied strains and in blue are root cells stained with calcofluor white.

The strains belonging to the genera *Bacillus* and *Paenibacillus* found by the immunolocalization technique showed rather discreet superficial colonization. *Bacillus altitudinis* VMFR45 and *Paenibacillus* sp. VMFR46 formed similar colonization patterns, with three-dimensional structures on the root surface that included the root hairs inside (Figure 7a,c). In both cases, they were distributed along with the intercellular spaces of the epithelial cells of the root, and characteristic geometric patterns were derived from this colonization (Figure 7b,d). *Paenibacillus amylolyticus* VMSES74′s colonization was more localized and discreet than that observed in the two previous strains, forming microcolonies on the root surface of the blueberry root (Figure 7e). In this case, cells were located in the valleys formed at the junction of the root epidermis cells (Figure 7f).

## 4. Discussion

*V. myrtillus* is a dwarf shrub of the *Ericaceae* family characterized by its growth in humid and temperate or cold climates. Its southernmost distribution occurs mainly in mountainous or high mountain regions, such as in the Iberian Peninsula [31]. Their theoretical ecological limit is restricted by the mountains in the north of the peninsula, mainly characterized by a humid climate and Atlantic influence [5]. However, in the mountain systems of the center and south of the peninsula, it appears as a relic of the glacial period [8]. To date, there has been no study of this plant in this region. Indeed, most of the published studies only focused on boreal and northern regions [15,32] or were conducted on other species of the genus *Vaccinium* [7,14,33]. Owing to their membership in the *Ericaceae* family, most works have focused on communities of mycorrhizal fungi since these create a specific formation with great relevance for their ecology due to the contribution of nutrients [15,16,34,35]. Relative to other studies, the analysis of rhizospheric and endophytic communities of blueberries from Finland showed that the rhizospheric communities had a similar composition to Acidobacteria, Actinobacteria, and Proteobacteria; however, at the endophytic level, the Finnish isolated communities did not show the dynamics observed in this study [32]. Indeed, in isolated Portuguese communities, the proportion of Actinobacteria was higher than that of Proteobacteria, while in those from Finland, the reverse was observed. In another work, it was observed that the phylum Planctomycetes was present only in the rhizospheric communities [15]. A similar community pattern was observed in other species of the genus *Vaccinium*, such as *V. angustifolium*, a species native to North America, in which the rhizospheric communities are mainly composed of Acidobacteria, Actinobacteria, and Proteobacteria; even so, some communities show peculiarities related to the age of the plant or the population, varying the predominance of Proteobacteria [14]. On the other hand, the analysis of communities on the fruiting and flowering surfaces of *V. macrocarpum* indicated the presence of heterogeneous communities. Here, the phyla Proteobacteria was predominant, dominated by different factors from those applicable to rhizo-endophytic environments [33]. The alpha diversity determined in this study is slightly higher than in other species of the genus *Vaccinium* (Figure 3), with up to 1.5 points difference in the Shannon–Wiener diversity index compared to the crop communities [14]. On the other hand, this study found that rhizospheric communities of blueberry associated with understory (Serra do Marão) or humid scrub (Serra da Freita) had similar diversity indices (Figure 3) and compositions to individual taxa detected upstream of Serra da Estrela populations (periglacial peat bog environments). The obtained data are consistent with other studies that showed that rhizospheric communities are influenced not only by the roots that dominate the rhizosphere but also by the heterogeneity of the canopy [36]. 

Numerous articles have also shown that, although the plant microbiome can vary according to environmental or edaphoclimatic factors, such as precipitation, soil composition, or temperature, plants are able to regulate the microorganisms that colonize their interior [37,38]. Thus, the plant can select the bacteria that it wants to interact with, exerting an important influence on its rhizosphere and especially on the endosphere [39]. Indeed, as we have seen in this work, the endophytic populations have a similar composition and structure, which limits the invasion of some bacterial orders (Figure 2). 

Therefore, the study of these microbiomes allows for the development of new agronomic strategies. Moreover, their study helps us to understand how the accessory microbiome improves plant plasticity, such as in Portuguese blueberries, where the climate limits their development [40,41,42,43]. As observed in the present study, these plants may have an influence on the rhizospheric bacterial communities, in which Actinobacteria, Acidobacteria, and Proteobacteria are core microbial communities, and other variable phyla occur depending on the isolation of the region. This has also been observed in other plants, such as *Arabidopsis*, where the production of exudates altered the structure of rhizospheric populations; even so, this change also depends on the age and developmental stage of the plant [44]. Similar effects have been observed in rice, wheat, pea, and sugarcane plants, where the plant exerts a modulating effect on surrounding populations, favoring certain taxa over others [45]. 

Although knowledge of the communities that make up the plant microbiome can provide us with important information about their biology and relationships with the environment, the search for and development of bacterial biofertilizers inevitably involves the cultivation and manipulation of microorganisms. Therefore, laboratory studies may be difficult due to the physiological needs of microorganisms adapted to environmental conditions, which are more restrictive in terms of nutrients [45,46]. In the present work, five different culture media were used to cover the widest possible range of culture conditions and not introduce factors that could limit their use in the production of biofertilizers [47]. Thus, the development of biofertilizers goes beyond the cultivation and knowledge of culturomes. In fact, large amounts of information are generated annually through the study of metagenomes but not cultivated bacteria, so it is necessary to create collections of cultivated strains to determine their ability as good biofertilizers [45]. In this regard, knowledge about the blueberry culturome is very limited. Thus far, a study conducted on endophytic populations of *V. myrtillus* leaves from Nordic regions showed that the genera *Bacillus* and *Paenibacillus* are frequently isolated [32], accounting for up to 20% of endophytic isolates at some sites (Figure 4). Moreover, that research also isolated strains identified in the genera *Staphylococcus* or *Pseudomonas* but not *Arthrobacter*, *Microbispora*, and *Streptomyces*, as in the present work. Thus, these data indicate that the selection of the strains of these phyla depends on the biome. In turn, a low number of pathogenic bacteria was detected compared to other studies that highlighted the presence of pathogenic bacteria as part of the microbiota of crops, such as rice and wheat [48,49]. The use of MALDI-TOF added value and proved to be a highly plausible tool for the identification of bacterial populations from the environment due to the inherent characteristics of the technique (speed, repeatability, low cost) [50], especially for the rapid detection of human pathogens with a high degree of accuracy [51]. In addition, it can be useful in the selection of microorganisms considered as biofertilizers among environmental bacteria. Studying environmental bacteria provides a high level of biosafety with a minor risk of selecting strains with pathogenic potential [52].

Analysis of PGP mechanisms among the 142 isolates showed that the PGP mechanism was homogeneously distributed among isolates, with some more common mechanisms, such as the solubilization of bicalcium phosphate or IAA production. Regarding the solubilization of phosphate, the data are in agreement with the literature, which indicates that the screening of this ability must be performed with several phosphate sources. Tricalcium phosphate and hydroxyapatite sources show more restrictive values, and bicalcium phosphate can produce false positive results due to its hydrolyzability by weak acids [53,54]. For instance, some genera that have been found to have a high ability to solubilize phosphate, such as *Pseudomonas*, have been described as good PGP microorganisms due to their ability to solubilize inorganic phosphate [55,56]. In recent years, the genus *Caballeronia*, with various strains isolated from the rhizosphere or endophytic environments, has been shown to be an important phosphate solubilizer [57,58,59]. Moreover, the production of siderophores has also been studied and demonstrated in about half of the isolates. Although this is not likely to be important for the uptake of iron by blueberry plants, which grow in acidic soil, where iron has high solubility and availability to the plant [60], this feature may be important for aspects of biocontrol and the management of soil iron stocks and its availability to phytopathogenic organisms [61,62]. Regarding the production of IAA, this mechanism is widespread among microorganisms from the rhizosphere and endophytic environments; however, production of IAA varies depending on the genus studied [63,64]. Discrete producers of this phytohormone are desirable because its overproduction is associated with plant pathogenic species [65,66]. Additionally, we can highlight the observed variability between spectrometric quantification methods and chromatographic methods used for the detection of this phytohormone (Table 1 and Table 2). Comparing both techniques, spectrometric ones are commonly used in screening the production of this phytohormone [67,68,69,70], allowing for the analysis of a large number of strains. However, the obtained values are not accurate in contrast to the use of chromatographic techniques, such as HPLC-DAD, used in this report, which permitted the quantification of IAA concentration without the interference of other derived indoles [71,72,73].

Regarding the interaction in the plant, studies focused on the analysis of the effects of the application of mycorrhizal fungi in different species of the genus *Vaccinium* are common [15,74,75]. Additionally, only a few articles have analyzed the inoculation of bacteria to promote blueberry plant development. The incorporation of *Bacillus toyonensis* COPE52 has already shown the potential to improve aerial and root development, as well as the concentration of chlorophyll [76]. It has also been described that the combined use of microorganisms in commercial preparations can have a positive effect; in particular, it was reported that the use of the genera *Bacillus*, *Pseudomonas*, *Nocardia*, *Saccharomyces*, and *Trichoderma* increased aerial development by almost 40% [77]. In the present work, we reported the efficacy of some strains of the genera *Caballeronia* and *Paraburkholderia*, both derived from the genus *Burkholderia*, distinguishing between species with an environmental character and those with a clinical character and pathogenic capabilities, currently assigned to the genus *Burkholderia* [78,79]. Thus, the obtained data confirm that both genera may be excellent candidates for the selection of bacteria with the potential to enhance crop development and production, particularly in blueberries, as has already been observed in grapevines [80], strawberries [81], some legumes [82], reeds [59], and tomatoes [58]. 

To date, studies on blueberry roots’ colonization have only focused on the behavior of mycorrhizal fungi (ericoid mycorrhizae) and dark septate endophytes (DSE) fungi [15,83]. The analysis of colonization capacity is an excellent way to predict how bacteria will interact with a host plant [84]. Thus, the colonization patterns observed in our strains are comparable to those observed in similar bacteria in other plants, such as *Paenibacillus* and *Bacillus* in wheat and tomato [85], or *Paenibacillus* in maize [86]. In the present study, the colonization patterns observed in strains *Caballeronia* sp. VMSES31 and *Paraburkholderia* sp. VMFR74 were similar to those observed in other strains of the genus *Paraburkholderia* when they colonized the rhizoplanes of ryegrass, tomato, sugarcane, barley, sorghum, and wheat [87,88]. Furthermore, these data show that the behavior of the different bacteria can be modified by molecules produced by the plant itself [89,90,91].

## 5. Conclusions

This work has shown that the bacterial populations of blueberries in Portugal are determined by the influence of the plant. In fact, although the rhizospheric communities are determined by environmental conditions, the endophytic communities exhibit a high degree of selection by the plant, having a similar composition and beta diversity. Thus, these data support the idea that plants select the microbiome beyond the edaphoclimatic conditions under which they evolve. These communities are a source of bacteria, with the PGP potential able to enhance plant development. The obtained results also reveal that some genera, such as *Bacillus*, *Paenibacillus*, and *Pseudomonas*, are important candidates for the selection of new biofertilizers, while other genera, such as *Caballeronia*, have significant root colonization ability. Finally, it is clear that the culturomic study of bacterial communities associated with the rhizobiome and plant endobiome is essential to isolate new strains with the potential to be considered multifunctional biofertilizers and for little-studied crops, such as blueberries.

## Figures and Tables

**Table 1:** PGP mechanisms of the selected strains and quantification of IAA by HPLC-DAD.

Strain	Bicalcium Phosphate (PSI)	Tricalcium Phosphate (PSI)	Hydroxyapatite (PSI)	Siderophore	IAA (µg/mL)
VMSES02				-	2.14 ± 0.02
VMSES14	0.90		0.75	-	1.62 ± 0.01
VMSES 32	0.69			+	4.67 ± 0.02
VMSES31	2.53		1.91	+	1.30 ± 0.01
VMSES36		0.21		-	0.52 ± 0.02
VMSES63				+	0.76 ± 0.16
VMSES74				+	8.81 ± 0.02
VMFR04B	0.55			+	12.35 ± 0.09
VMFR45	0.46	0.39		+	2.80 ± 0.02
VMFR46	0.60	0.23		+	0.77 ± 0.01
VMFR53				+	1.17 ± 0.02
VMMAR66				+	1.89 ± 0.02
Mirt15	1.02	1.05	0.35	+	38.37 ± 0.08
Mirt26				+	1.18 ± 0.11
Mirt39A	1.45	1.20	0.31	+	26.57 ± 0.02

PSI: Phosphate solubilization index resulted from solubilization halum–colony halum.

**Table 2:** Growth parameters of tomato and blueberry seedlings inoculated with the selected strains.

	Tomato 7 dpi			Tomato 14 dpi			Blueberry 7 dpi			Blueberry 14 dpi		
Treatment	Root Length (cm)	Secondary roots	Aerial Length (cm)	Root Length (cm)	Secondary roots	Aerial Length (cm)	Root Length (cm)	Secondary roots	Aerial Length (cm)	Root Length (cm)	Secondary roots	Aerial Length (cm)
Control	1.80 ± 0.14a	2.60 ± 0.41a	2.77 ± 0.32b	3.50 ± 0.44a	4.01 ± 0.60b	3.70 ± 0.48b	0.91 ± 0.24a	2.00 ± 0.50a	0.39 ± 0.10a	1.32 ± 0.11a	3.21 ± 0.75a	0.62 ± 0.32a
MIRT15	2.47 ± 0.51a	1.83 ± 0.64a	1.80 ± 0.46a	3.73 ± 0.78a	3.03 ± 0.96 a	2.68 ± 0.68a	0.96 ± 0.17a	2.00 ± 0.50a	0.41 ± 0.07a	1.38 ± 0.10a	2.83 ± 1.00a	1.02 ± 0.41ab
MIRT26	2.47 ± 0.46a	2.20 ± 0.72a	1.81 ± 0.43a	3.71 ± 0.68a	3.10 ± 1.08 a	2.71 ± 0.64a	0.87 ± 0.44a	2.00 ± 0.50a	0.44 ± 0.07a	1.42 ± 0.09a	3.01 ± 1.15a	0.74 ± 0.22a
MIRT39A	2.10 ± 0.50a	2.01 ± 0.56a	3.22 ± 0.45b	3.10 ± 0.76a	3.02 ± 0.84 a	5.20 ± 0.67c	1.02 ± 0.16a	2.00 ± 0.50a	0.43 ± 0.05a	1.26 ± 0.20a	2.87 ± 0.95a	0.77 ± 0.36a
VMFR04B	3.40 ± 0.38b	6.01 ± 1.46bc	3.11 ± 0.37b	5.10 ± 0.57b	9.09 ± 2.21d	4.78 ± 0.56c	1.05 ± 0.41a	2.00 ± 0.10a	0.41 ± 0.02a	2.06 ± 0.13b	2.71 ± 0.74a	1.18 ± 0.34ab
VMFR45	3.92 ± 0.43b	6.10 ± 1.20bc	3.77 ± 0.29c	6.11 ± 0.68c	9.11 ± 1.79d	5.71 ± 0.56c	1.00 ± 0.43a	2.00 ± 0.30a	0.43 ± 0.04a	2.01 ± 0.19b	2.89 ± 0.72a	1.29 ± 0.21b
VMFR46	3.80 ± 0.40b	6.02 ± 1.41bc	3.67 ± 0.46c	6.12 ± 0.60c	9.01 ± 2.12d	5.88 ± 0.68c	0.96 ± 0.22a	2.00 ± 0.20a	0.42 ± 0.09a	2.16 ± 0.18b	2.95 ± 0.52a	1.42 ± 0.21b
VMFR53	3.60 ± 0.43b	4.60 ± 0.56b	3.27 ± 0.41b	5.41 ± 0.64b	7.04 ± 0.84c	4.92 ± 0.60c	1.10 ± 0.16a	3.00 ± 0.20b	0.45 ± 0.07a	1.97 ± 0.17b	3.21 ± 0.74a	1.32 ± 0.37b
VMMAR66	3.27 ± 0.29b	4.00 ± 1.27ab	2.62 ± 0.28b	4.89 ± 0.44b	6.21 ± 1.90c	3.91 ± 0.42b	0.93 ± 0.44a	2.00 ± 0.50a	0.44 ± 0.051a	2.01 ± 0.21b	2.66 ± 0.12a	1.11 ± 0.28ab
VMSES02	3.20 ± 0.49ab	7.31 ± 1.18c	2.33 ± 0.44ab	4.78 ± 0.73b	11.13 ± 1.92d	3.58 ± 0.67b	0.97 ± 0.42a	2.00 ± 0.20a	0.42 ± 0.03a	1.99 ± 0.07b	2.34 ± 0.88a	1.09 ± 0.51ab
VMSES14	3.50 ± 0.35b	4.30 ± 0.97b	3.44 ± 0.48bc	5.22 ± 0.73b	7.32 ± 1.45c	5.51 ± 0.67c	0.88 ± 0.21a	3.00 ± 0.50b	0.42 ± 0.07a	2.03 ± 0.14b	3.47 ± 0.62a	1.32 ± 0.42b
VMSES31	3.80 ± 0.49b	5.30 ± 0.65b	2.76 ± 0.45b	5.71 ± 0.74bc	8.04 ± 0.98cd	4.21 ± 0.66b	1.02 ± 0.41a	2.00 ± 0.20a	0.41 ± 0.07a	1.85 ± 0.14b	2.73 ± 0.65a	1.27 ± 0.46ab
VMSES32	3.00 ± 0.41ab	2.60 ± 0.74a	3.22 ± 0.43b	5.69 ± 0.69bc	4.17 ± 1.12b	4.80 ± 0.64c	0.89 ± 0.2a	2.00 ± 0.20a	0.52 ± 0.06a	1.89 ± 0.21b	2.37 ± 0.62a	1.12 ± 0.61ab
VMSES63	3.40 ± 0.34b	5.10 ± 1.01b	3.13 ± 0.44b	5.09 ± 0.76b	9.27 ± 1.63c	4.71 ± 0.66c	1.14 ± 0.16a	2.00 ± 0.20a	0.43 ± 0.06a	1.95 ± 0.08b	2.62 ± 0.32a	1.28 ± 0.14b
VMSES74	3.62 ± 0.45b	4.61 ± 0.56b	3.07 ± 0.43b	5.60 ± 0.68bc	7.31 ± 0.84c	4.59 ± 0.64bc	0.87 ± 0.42a	3.00 ± 0.30b	0.42 ± 0.062a	1.99 ± 0.16b	3.20 ± 0.84a	0.98 ± 0.32a

Means (*n* = 30) are shown. Treatment means are classified into groups according to Tukey comparison tests at *p* ≤ 0.0001.

## Data Availability

The raw sequences from the metabarcoding experiment have been deposited with the BioProject ID PRJNA786857, SRA numbers SRX13339950 to SRX13339967 (https://www.ncbi.nlm.nih.gov/bioproject/?term=PRJNA786857, accessed on 10 October 2022). The data-sets generated and analyzed during the current study are included in this published article (and its Supplementary Information files).

## Supplementary Materials

The following supporting information can be downloaded at: https://www.mdpi.com/article/10.3390/microorganisms10112193/s1, Table S1: Sequencing statistic for each sample; Table S2: Isolated strains, identification based on MALDI-TOF and 16S rDNA sequence and summary of PGP mechanisms.
