# Supplementary material for: Insight into the Taxonomic and Functional Diversity of Bacterial Communities Inhabiting Blueberries in Portugal
Source: Microorganisms. 2022 Nov 4;10(11):2193. doi: 10.3390/microorganisms10112193 (PMC9695653; doi:10.3390/microorganisms10112193)
Supplement: Supplementary file 1 [file microorganisms-10-02193-s001.zip › supplementary Table S1.pdf]

Table S1. Sequencing statistic for each sample

Sample ID	Sample type	Sample location	DNA concentration (ng/μL)	Raw reads*	Filtered reads	Denoised reads	Merged reads	Non-chimeric sequences
**EST1**	Rhizospheric soil	Serra da Estrela	60.4	106245	87770	73719	51231	48714
**EST2**	Rhizospheric soil	Serra da Estrela	30.6	128069	105114	90151	66399	63405
**EST4**	Rhizospheric soil	Serra da Estrela	37.8	87863	71536	59300	41577	39745
**FR1**	Rhizospheric soil	Serra da Freita	34.4	135804	112428	93026	61413	57417
**FR2**	Rhizospheric soil	Serra da Freita	35.2	105161	86668	70254	45597	42786
**FR3**	Rhizospheric soil	Serra da Freita	22.4	98780	80915	66278	43575	41211
**MAR1**	Rhizospheric soil	Serra de Marao	32	97832	79015	65351	41994	40025
**MAR2**	Rhizospheric soil	Serra de Marao	42.2	84072	68836	54872	35007	33673
**MAR3**	Rhizospheric soil	Serra de Marao	54.2	107605	88063	71552	45592	43151
**REST1**	Vaccinium myrtilus roots	Serra da Estrela	26.6	77529	63881	59593	52541	46453
**REST2**	Vaccinium myrtilus roots	Serra da Estrela	41.8	90647	75401	70219	61547	53649
**REST3**	Vaccinium myrtilus roots	Serra da Estrela	33.2	105753	87704	81994	71969	62082
**RFR1**	Vaccinium myrtilus roots	Serra da Freita	17	104603	86748	81186	71711	61824
**RFR2**	Vaccinium myrtilus roots	Serra da Freita	20.2	103191	86081	80417	70705	61171
**RFR3**	Vaccinium myrtilus roots	Serra da Freita	14.9	122313	100449	94298	84318	73407
**RMAR1**	Vaccinium myrtilus roots	Serra de Marao	11.3	95729	78150	73505	65509	58610
**RMAR2**	Vaccinium myrtilus roots	Serra de Marao	16.6	88006	72920	68312	61087	53843
**RMAR3**	Vaccinium myrtilus roots	Serra de Marao	9.2	75238	58000	54196	48210	43765
**BPCR ♣**				115	51	11	8	8

Sample IDs, Qubit quantification values, and number of raw reads and sequences obtained per sample. * Number of raw reads from paired-end sequencing, i.e., forward (R1) reads or reverse (R2) reads. ♣ PCR negative controls (BPCR) reads.
