# Supplementary material for: Insight into the Taxonomic and Functional Diversity of Bacterial Communities Inhabiting Blueberries in Portugal
Source: Microorganisms. 2022 Nov 4;10(11):2193. doi: 10.3390/microorganisms10112193 (PMC9695653; doi:10.3390/microorganisms10112193)
Supplement: Supplementary file 1 [file microorganisms-10-02193-s001.zip › supplementary Table S2.pdf]

Table S2. Isolated strains, identification based on MALDI-TOF and 16S rDNA sequence and summary of PGP mechanisms.

Strain	Location	Source	MALDI TOF		rRNA 16S		Accesion number	PGP mechanisms				
			Identity	score	Identity	% similarity		BiP	TriP	HXP	SID	IAA
VMSES01	Serra da Estrela	Soil	*Bacillus cereus*	2.219	*Bacillus toyonensis* BCT-7112T	99,87	MZ234580.1					85,39
VMSES02	Serra da Estrela	Soil	*Not reliable identification*	1.442	*Psychrobacillus glaciei* PB01T	99,74	MZ234581.1					5,22
VMSES03	Serra da Estrela	Soil	*Not reliable identification*	1.341	*Bacillus bataviensis* IDA1115T	98,63	MZ234582.1					103,63
VMSES04	Serra da Estrela	Soil	*Not reliable identification*	1.498	*Paenibacillus lutimineralis* MBLB1234T	99.06	OM963150.1	0,118				22,61
VMSES05	Serra da Estrela	Soil	*Bacillus novalis*	1.802	*Neobacillus novalis* IDA3307T	99,73	OM963145.1					
VMSES06	Serra da Estrela	Root	*Bacillus muralis*	1.736	*Bacillus muralis* LMG 20238T	99,66	MZ234583.1					0,98
VMSES07	Serra da Estrela	Root	*Not reliable identification*	1.490	*Cytobacillus luteolus* YIM 93174T	99,39	MZ234584.1	0,163				1,82
VMSES08	Serra da Estrela	Root	*Bacillus novalis*	2.289	*Neobacillus novalis* IDA3307T	99,66	OM963146.1	0,160				3,10
VMSES11	Serra da Estrela	Root	*Bacillus novalis*	2.067	*Neobacillus novalis* IDA3307T	99,66	OM963147.1					18,79
VMSES12	Serra da Estrela	Soil	*Not reliable identification*	1.571	*Mesobacillus maritimus* KS16-9T	98,47	MZ234585.1					5,22
VMSES13	Serra da Estrela	Soil	*Paenibacillus amylolyticus*	2.291	*Paenibacillus amylolyticus* NRS-290T	99,07	OM963148.1					18,79
VMSES14	Serra da Estrela	Soil	*Pseudomonas frederiksbergensis*	1.956	*Pseudomonas frederiksbergensis* DSM 13022T	99,61	MZ234586.1	0,905		0,754		32,79
VMSES21	Serra da Estrela	Soil	*Pseudomonas koreensis*	1.914	*Pseudomonas tolaasii* LMG 2342T	99,93	MZ234587.1					66,30
VMSES22	Serra da Estrela	Soil	*Pseudomonas koreensis*	1.966	*Pseudomonas brassicaceraum* ATCC 49054T	99,58	OM670212.1	0,140				62,06
VMSES25A	Serra da Estrela	Soil	*Staphylococcus capitis*	2.449								
VMSES25B	Serra da Estrela	Soil	*Pseudomonas koreensis*	2.151	*Pseudomonas synxantha* NCTC10696T	99,20	MZ234588.1	0,345				54,00
VMSES26	Serra da Estrela	Soil	*Staphylococcus capitis*	2.384								
VMSES27B	Serra da Estrela	Soil	*Pseudomonas koreensis*	2.304				0,045				
VMSES28A	Serra da Estrela	Soil	*Bacillus bataviensis*	2.029	*Bacillus bataviensis* NBRC 102449T	98,98	OM963142.1					
VMSES28B	Serra da Estrela	Soil	*Bacillus bataviensis*	1.877	*Bacillus bataviensis* NBRC 102449T	98,98	MZ234589.1					
VMSES 32	Serra da Estrela	Root	*Pseudomonas koreensis*	2.308				0,695			+	37,46
VMSES30	Serra da Estrela	Root	*Roseomonas mucosa*	1.869	*Roseomonas mucosa* MDA5527T	99,72	OM963149.1	0,100			+	33,21
VMSES31	Serra da Estrela	Root	*not reliable identification*	1.589	*Paraburkholderia domus* LMG 31832T	99,93	OM963143.1	2,529		1,906	+	31,52
VMSES36	Serra da Estrela	Root	*not reliable identification*	1.513	*Paraburkholderia sediminicola* HU2-65WT	99,93	MZ234590.1		0,212			
VMSES37	Serra da Estrela	Root	*not reliable identification*	1.258	*Luteibacter rhizovicinus* LJ96T	99,53	MZ234591.1	0,306			+	
VMSES38	Serra da Estrela	Root	*not reliable identification*	1.534	*Paenibacillus dokdohensis* T	98,18	OM963155.1		0,212			
VMSES39	Serra da Estrela	Root	*not reliable identification*	1.326	*Luteibacter rhizovicinus* LJ96T	99,86	MZ234592.1	0,588	1,034		+	3,95
VMSES40	Serra da Estrela	Root	*not reliable identification*	1.660	*Aquaspirillum articum* IAM 14963T	99,73	MZ234593.1					191,44
VMSES43	Serra da Estrela	Root	*Not reliable identification*	1.647	*Pseudomonas silesensis* A3T	99,53	MZ234594.1					
VMSES46	Serra da Estrela	Root	*Not reliable identification*	1.465	*Nevskia soli* GR15-1T	99,93	MZ234595.1		0,245		+	17,10
VMSES47	Serra da Estrela	Root	*Bacillus muralis*	1.795	*Steptomyces hundungensis* MBRL 251T	100	MZ234596.1	0,702	0,621			
VMSES48	Serra da Estrela	Soil	*Candida zeylanoides*	1.774								
VMSES49	Serra da Estrela	Soil	*not reliable identification*	1.430	*Bacillus muralis* LMG 20238T	99,59	MZ234597.1					

VMSES50	Serra da Estrela	Soil	*not reliable identification*	1.577	*Collimonas antrihumi* C3-17T	99,86	OM963141.1					
VMSES51	Serra da Estrela	Soil	*not reliable identification*	1.675	*Peribacillus muralis* LMG 20238T	99,13	MZ234598.1	0,196		0,100	+	19,22
VMSES52	Serra da Estrela	Soil	*Burkholderia xenovorans*	1.706	*Shigella flexneri* ATCC 29903T	99,52	MZ234599.1					
VMSES53	Serra da Estrela	Soil	*not reliable identification*	1.451	*Rugamonas rivuli* FT103WT	98,61	MZ234600.1				w	267,37
VMSES56	Serra da Estrela	Soil	*not reliable identification*	1.576	*Pseudomonas frederiksbergensis* DSM 13022T	99,8	MZ234601.1	1,133			+	
VMSES57	Serra da Estrela	Root	*not reliable identification*	1.562	*Streptomyces hundungensis* MBRL 251T	99,38	ON585117.1	0,333	1,333		+	10,31
VMSES59	Serra da Estrela	Root	*Not reliable identification*	1.490	*Duganella phyllosphaerae* FT103WT	98,44	MZ234602.1					
VMSES60	Serra da Estrela	Root	*Bacillus soli*	2.068	*Neobacillus drentensis* NBRC 102427T	99,57	OM670213.1				+	23,03
VMSES64	Serra da Estrela	Root	*not reliable identification*	1.542	*Paenibacillus odorifer* DSM 15391T	99,54	OM670214.1				+	
VMSES63	Serra da Estrela	Root	*not reliable identification*	1.558	*Paenibacillus chibensis* JCM 9905T	96,75	MZ234603.1				+	
VMSES70	Serra da Estrela	Root	*not reliable identification*	1.484	*Paraburkholderia metrosideri* ATCC BAA-463T	98,59	MZ234605.1		0,032		+	36,61
VMSES66	Serra da Estrela	Root	*not reliable identification*	1.577	*Caballeronia udeis* Hg2T	98,95	MZ234604.1				+	
VMSES74	Serra da Estrela	Root	*not reliable identification*	1.522	*Paenibacillus amylolyticus* NRRL NRS-290T	99,27	OM670215.1	0.112			+	
VMFR01	Serra da Freita	Root	*Not reliable identification*	1.430	*Bacillus sporothermodurans* DSM 10599T	99,46	MZ234606.1				+	6,49
VMFR02	Serra da Freita	Soil	*Bacillus cereus*	1.920	*Bacillus mobilis* MCCC 1A05942T	99,93	MZ234607.1				+	10,73
VMFR03	Serra da Freita	Soil	*Viridibacillus arvi*	2.169	*Buttiauxella noackiae* NSW 11T	99,43	MZ234608.1				+	87,51
VMFR04A	Serra da Freita	Soil	*Bacillus mycoides*	2.281	*Bacillus mycoides* ATCC 6462T	99,93	MZ234609.1				+	
VMFR04B	Serra da Freita	Soil	*Bacillus pumilus*	1.911	*Bacillus aerius* 24KT	99,77	MZ234610.1	0,554			+	87,94
VMFR05	Serra da Freita	Soil	*Paenibacillus pabuli*	2.276	*Paenibacillus pabuli* JCM 9074T	99,5	MZ234611.1				+	135,02
VMFR08	Serra da Freita	Soil	*Bacillus mycoides*	2.038	*Bacillus nakamurai* NRRL B-41091T	100	MZ234612.1				+	27,28
VMFR09	Serra da Freita	Soil	*Bacillus pumilus*	2.111	*Bacillus australimaris* MCCC 1A05787T	99,87	MZ234613.1	0,381			+	146,05
VMFR10	Serra da Freita	Soil	*Bacillus mycoides*	2.055	*Bacillus mycoides* ATCC 6462T	99,79	MZ234614.1	0,254			+	32,37
VMFR11	Serra da Freita	Soil	*Bacillus cereus*	2.323				0,258			+	159,20
VMFR12	Serra da Freita	Soil	*Bacillus subtilis*	1.943	*Bacillus nakamurai* NRRL B-41091T	99,67	MZ234615.1				+	94,30
VMFR13	Serra da Freita	Soil	*Bacillus mycoides*	2.268	*Bacillus mycoides* ATCC 6462T	100	MZ234616.1				+	
VMFR14	Serra da Freita	Soil	*Bacillus mojavensis*	2.042	*Bacillus velezensis* NRRL B-41580T	99,73	MZ234617.1				+	197,80
VMFR17A	Serra da Freita	Soil	*Paenibacillus alvei*	1.724	*Paenibacillus alvei* NBRC 3343T	98,92	MZ234618.1				+	18,37
VMFR18	Serra da Freita	Soil	*Bacillus mojavensis*	1.964	*Bacillus velezensis* NRRL B-41580T	99,8	MZ234619.1				+	202,89
VMFR19	Serra da Freita	Soil	*Bacillus cereus*	2.354	*Bacillus paraanthracis* MCCC 1A00395T	99,55	OM670216.1	0,167			+	55,27
VMFR20	Serra da Freita	Soil	*Buttiauxella gaviniae*	2.006	*Buttiauxella noackiae* NSW 11T	99,16	MZ234620.1	0,672				114,24
VMFR21	Serra da Freita	Soil	*Serratia quinivorans*	2.043								
VMFR23	Serra da Freita	Root	*Not reliable identification*	1.347	*Microbispora bryophytorum* NEAU-TX2-2T	99,59	MZ234622.1	1,049				
VMFR25	Serra da Freita	Root	*Not reliable identification*	1.465	*Microbispora bryophytorum* NEAU-TX2-2T	99,19	OM670217.1					126,54
VMFR27	Serra da Freita	Soil	*Serratia liquefaciens*	2.014								
VMFR28A	Serra da Freita	Root	*Staphylococcus capitis*	2.405								
VMFR28B	Serra da Freita	Root	*Staphylococcus capitis*	2.445								

VMFR30	Serra da Freita	Root	*Staphylococcus capitis*	2.310								
VMFR33	Serra da Freita	Root	*Not reliable identification*	1.641	*Microbispora catharanthi* CR1-09T	100	OM670219.1					
VMFR34	Serra da Freita	Root	*Not reliable identification*	1.522	*Microbispora bryophytorum* NEAU-TX2-2T	99,6	MZ234624.1					12,00
VMFR35	Serra da Freita	Root	*Not reliable identification*	1.618	*Conhella saccharovorans* CJ22T	97,36	MZ234625.1					13,70
VMFR36	Serra da Freita	Root	*Serratia liquefaciens*	1.722	*Shigella flexneri* ATCC 29903T	99,65	MZ234626.1					
VMFR37	Serra da Freita	Root	*Not reliable identification*	1.432	*Sphaerimonospora mesophila* JCM 3151T	99,52	MZ234627.1					44,24
VMFR38	Serra da Freita	Root	*Not reliable identification*	1.674	*Escherichia coli* DSM 30083T	99,65	MZ234628.1					
VMFR39	Serra da Freita	Soil	*Pseudomonas chlororaphis*	1.977	*Pseudomonas eucalypticola* NP-1T	99,66	MZ234629.1	0,129				40,43
VMFR40	Serra da Freita	Soil	*Burkholderia phymatum*	1.926	*Paraburkholderia aromaticivorans* BN5T	99,8	MZ234630.1					28,55
VMFR41	Serra da Freita	Soil	*not reliable identification*	1.457	*Chryseobacterium piperi* ATCC BAA-1782T	99,65	MZ234631.1				+	24,73
VMFR42	Serra da Freita	Soil	*Burkholderia xenovorans*	1.957	*Paraburkholderia dipogonis* DL7T	100	MZ234632.1				+	43,40
VMFR43	Serra da Freita	Soil	*not reliable identification*	1.453	*Dyella koorensis* NBRC 100831T	99,86	MZ234633.1					13,28
VMFR45	Serra da Freita	Root	*not reliable identification*	1.657	*Bacillus altitudinis* 41KF2bT	100	MZ234634.1	0,459	0,393		+	13,70
VMFR46	Serra da Freita	Root	*Bacillus pumilus*	1.936	*Paenibacillus chibensis* JCM 9905T	96,47	MZ234635.1	0,603	0,234		+	12,85
VMFR48	Serra da Freita	Soil	*Bacillus pumilus*	1.974	*Bacillus altitudinis* 41KF2bT	99,93	OM670223.1				+	
VMFR51	Serra da Freita	Soil	*Burkholderia phymatum*	1.714	*Duganella levis* CY42WT	100	MZ234636.1	0,140	0,176		w	173,20
VMFR52	Serra da Freita	Soil	*not reliable identification*	1.454	*Paenibacillus dokdohensis* T	99,48	OM670224.1				+	
VMFR53	Serra da Freita	Soil	*not reliable identification*	1.457	*Caballeronia jiansuensis* MP-1T	100	MZ234637.1				+	0,55
VMFR59	Serra da Freita	Soil	*not reliable identification*	1.644	*Paraburkholderia ginsengisoli* NBRC 100965T	99,93	MZ234638.1		0,019			23,03
VMFR72	Serra da Freita	Root	*not reliable identification*	1.595	*Arthrobacter bambusae* THG-GM18T	99,71	OM963144.1					23,88
VMMAR01	Serra de Marão	Soil	*Serratia liquefaciens*	2.385								
VMMAR02	Serra de Marão	Soil	*Staphylococcus xylosus*	2.321								
VMMAR05	Serra de Marão	Soil	*Leifsonia aquatica*	1.966	*Leifsonia naganoensis* DB103T	99,93	MZ234639.1				+	64,61
VMMAR06B	Serra de Marão	Soil	*Rahnella aquatilis*	1.986	*Rahnella aceris* SAP-19T	99,05	MZ234640.1				+	-2,42
VMMAR07	Serra de Marão	Soil	*Bacillus pumilus*	1.998	*Bacillus aereus* 41KF2bT	100	MZ234641.1	0,213			+	57,82
VMMAR08	Serra de Marão	Soil	*Pantoea agglomerans*	2.357								
VMMAR09	Serra de Marão	Soil	*Serratia liquefaciens*	2.446								
VMMAR10A	Serra de Marão	Root	*Bacillus mojavensis*	2.037	*Bacillus siamensis* KCTC 13613T	99,93	MZ234642.1				+	32,37
VMMAR13	Serra de Marão	Root	*Not reliable identification*	1.662	*Rahnella contaminans* Lac-M11T	98,65	MZ234643.1	0,129			+	217,32
VMMAR18	Serra de Marão	Root	*Pantoea agglomerans*	2.356								
VMMAR24	Serra de Marão	Root	*Buttiauxella brennerae*	2.003	*Buttiauxella brennerae* S1/6-571T	99,53	MZ234644.1					235,56
VMMAR25	Serra de Marão	Root	*Pantoea agglomerans*	2.175	*Pantoea vagans* LMG 24199T	99,50	MZ234645.1					
VMMAR26	Serra de Marão	Soil	*Not reliable identification*	1.623	*Rahnella bruchi* FRB 226T	99,85	MZ234646.1	0,825		0,941		47,21
VMMAR27	Serra de Marão	Soil	*Not reliable identification*	1.456	*Luteimonas terrae* THG-MD21T	99,39	MZ234647.1				+	87,94
VMMAR29	Serra de Marão	Soil	*Not reliable identification*	1.617	*Comamonas testosteroni* EJ-4T	99,17	MZ234648.1				-	14,13
VMMAR33	Serra de Marão	Root	*Viridibacillus neidei*	2.341	*Viridibacillus arvi* LMG 22165T	100	OM670220.1				+	33,64

VMMAR35	Serra de Marão	Soil	*Rhodococcus erythropolis*	2.449									
VMMAR37	Serra de Marão	Soil	*Microbacterium testaceum*	2.064	*Luteimonas terrae* THG-MD21T	98,98	MZ234649.1	0,370					37,88
VMMAR39	Serra de Marão	Soil	*Pantoea agglomerans*	1.899	*Pantoea agglomerans* FDAARGOST	99,79	OM670221.1						
VMMAR41A	Serra de Marão	Soil	*Not reliable identification*	1.688	*Paenibacillus terrae* AM141T	99,92	MZ234650.1	0,268				+	169,81
VMMAR41B	Serra de Marão	Soil	*Enterobacter asburiae*	2.141									
VMMAR45A	Serra de Marão	Root	*Serratia fonticola*	2.124									
VMMAR45B	Serra de Marão	Root	*Not reliable identification*	1.505	*Paraburkholderia phytofirmans* PsJNT	99,4	OM670222.1					+	4,79
VMMAR55	Serra de Marão	Root	*not reliable identification*	1.471	*Acerihabitans arboris* SAP-6T	98,56	OM670225.1						
VMMAR56	Serra de Marão	Soil	*Burkholderia xenovorans*	2.184	*Paraburkholderia xenovorans* LB400T	99,72	OM963151.1					+	45,94
VMMAR58	Serra de Marão	Soil	*Erwinia billingiae*	2.194									
VMMAR60	Serra de Marão	Soil	*not reliable identification*	1.653	*Luteibacter rhizovicinus* DSM 16549T	99,65	OM963139.1						45,09
VMMAR63A	Serra de Marão	Soil	*not reliable identification*	1.465	*Paenibacillus chibensis* JCM 9905T	97,36	OM963140.1					+	
VMMAR66	Serra de Marão	Soil	*not reliable identification*	1.452	*Caballeronia sordidicola* S5-BT	98,96	OM670227.1	1,435	0,769	0,943		+	
VMMAR70	Serra de Marão	Soil	*not reliable identification*	1.456	*Paraburkholderia dipogononis* DL7T	99,65	MZ234651.1					+	32,37
VMMAR72	Serra de Marão	Root	*Paenibacillus amylolyticus*	2.225	*Paenibacillus amylolyticus* NRRL NRS-290T	99,13	OM670228.1					w	
VMMAR64	Serra de Marão	Root	*Burkholderia phymatum*	1.701	*Caballeronia terrestris* R-23321T	99,53	OM670226.1		0,508				14,55
Mirt02B	Serra da Estrela	Soil	*not reliable identification*	1.575	*Paraburkholderia sediminicola* HU2-65WT	99,73	MZ234653.1						26,85
Mirt02C_1	Serra da Estrela	Soil	*not reliable identification*	1.525	*Peribacillus muralis* LMG 20238T	99,13	MZ234654.1						31,94
Mirt4B	Serra da Estrela	Root	*not reliable identification*	1.528	*Peribacillus muralis* LMG 20238T	98,94	MZ234656.1						26,85
Mirt03	Serra da Estrela	Root	*not reliable identification*	1.653	*Neobacillus bataviensis* NBRC 102449T	97,91	MZ234655.1					w	
Mirt06	Serra da Estrela	Soil	*Bacillus mycoides*	2.343					0,475			+	41,27
Mirt08	Serra da Estrela	Root	*not reliable identification*	1.512	*Peribacillus muralis* LMG 20238T	99,19	MZ234657.1					+	1,82
Mirt09	Serra da Estrela	Root	*Bacillus psychrosaccharolyticus*	2.022	*Peribacillus psychrosaccharolyticus* NBRC 101233T	99,66	MZ234658.1					+	12,85
Mirt13	Serra da Estrela	Soil	*Achromobacter spanius*	2.112	*Achromobacter spanius* LMG 5911T	99,79	OM963152.1	0,948	0,542	0,321		+	96,42
Mirt14	Serra da Estrela	Soil	*Serratia liquefaciens*	2.122									
Mirt15	Serra da Estrela	Soil	*Pseudomonas antarctica*	2.083	*Pseudomonas salomonii* ICMP 14252T	100	OM963154.1	1,017	1,055	0,353		+	104,48
Mirt16	Serra da Estrela	Soil	*Serratia liquefaciens*	1.999	*Paenibacillus pini* JCM 16418T	99,38	MZ234659.1	0,382	1,102			+	32,37
Mirt20	Serra da Estrela	Soil	*not reliable identification*	1.561	*Lysinibacillus agricola* FJAT-51161T	99,06	MZ234660.1					+	107,45
Mirt25	Serra da Estrela	Root	*Rhodococcus erythropolis*	2.153									
Mirt26	Serra da Estrela	Root	*not reliable identification*	1.276	*Paenibacillus pini* JCM 16418T	99,31	MZ234662.1					+	16,67
Mirt30	Serra da Estrela	Soil	*Streptomyces badius*	1.965	*Streptomyces griseus* DSM 40236T	100	MZ234663.1					+	26,00
Mirt39A	Serra da Estrela	Soil	*Pseudomonas marginalis*	2.013	*Pseudomonas allii* MAFF 301514T	100	OM963153.1	1,452	1,200	0,310		+	64,61
Mirt63	Serra da Estrela	Soil	*Moraxella_sg_Moraxella osloensis*	2.088									
MBSES07	Serra da Estrela	Soil	*not reliable identification*	1.534	*Caballeronia sordidicola* S5-BT	99,66	OM670230.1					+	41,27
MBSES08	Serra da Estrela	Soil	*not reliable identification*	1.675	*Pseudomonas pratensis* MHJ-10JT	99,87	OM670231.1					+	28,97
MBSES16	Serra da Estrela	Soil	*not reliable identification*	1.465	*Caballeronia udeis* Hg2T	98,94	OM670232.1					+	45,09

MBSES17	Serra da Estrela	Soil	*not reliable identification*	1.617	*Enterobacter ludwigii* EN-119T	99,8	OM670233.1

BCP-Bicalcium phosphate, TCP-Tricalcium phosphate, HXP-Hydroxyapatite, SD: siderophore production IAA-Indole-3-acetic acid. Siderophore production expressed as +(positive) or nd (not detected). IAA is expressed in μg/mL.
